# Probiotic *Limosilactobacillus reuteri* KUB-AC5 decreases urothelial cell invasion and enhances macrophage killing of uropathogenic *Escherichia coli in vitro* study

**DOI:** 10.3389/fcimb.2024.1401462

**Published:** 2024-07-18

**Authors:** Arishabhas Tantibhadrasapa, Songbo Li, Songphon Buddhasiri, Chutikarn Sukjoi, Panupon Mongkolkarvin, Pattarapon Boonpan, Somsakul Pop Wongpalee, Prasobsook Paenkaew, Sawannee Sutheeworapong, Massalin Nakphaichit, Sunee Nitisinprasert, Michael H. Hsieh, Parameth Thiennimitr

**Affiliations:** ^1^ Department of Microbiology, Faculty of Medicine, Chiang Mai University, Chiang Mai, Thailand; ^2^ Key Laboratory of Tumor Immunopathology, Youjiang Medical University for Nationalities, Baise, China; ^3^ Research Center for Veterinary Biosciences and Veterinary Public Health, Chiang Mai University, Chiang Mai, Thailand; ^4^ Faculty of Veterinary Medicine, Chiang Mai University, Chiang Mai, Thailand; ^5^ Pilot Plant Development and Training Institute (PDTI), King Mongkut’s University of Technology Thonburi (KMUTT), Bangkok, Thailand; ^6^ Department of Biotechnology, Faculty of Agro-Industry, Kasetsart University, Bangkok, Thailand; ^7^ Specialized Research Unit: Probiotics and Prebiotics for Health, Faculty of Agro-Industry, Kasetsart University, Bangkok, Thailand; ^8^ Department of Urology, School of Medicine and Health Sciences, The George Washington University, Washington, DC, United States; ^9^ Department of Pediatrics, School of Medicine and Health Sciences, The George Washington University, Washington, DC, United States; ^10^ Department of Microbiology, Immunology, and Tropical Medicine, School of Medicine and Health Sciences, The George Washington University, Washington, DC, United States; ^11^ Research Center of Microbial Diversity and Sustainable Utilization, Chiang Mai University, Chiang Mai, Thailand; ^12^ Center of Multidisciplinary Technology for Advanced Medicine, Faculty of Medicine, Chiang Mai University, Chiang Mai, Thailand

**Keywords:** uropathogenic *E. coli* (UPEC), urinary tract infection (UTI), probiotic, *Limosilactobacillus reuteri*, immune modulation, immunobiotic

## Abstract

**Introduction:**

Bacterial urinary tract infections (UTI) are among the most common infectious diseases worldwide. The rise of multidrug-resistant (MDR) uropathogenic *Escherichia coli* (UPEC) UTI cases is a significant threat to healthcare systems. Several probiotic bacteria have been proposed as an alternative to combat MDR UTI. Lactic acid bacteria in the genus *Limosilactobacillus* are some of the most studied and used probiotics. However, strain-specific effects play a critical role in probiotic properties. *L. reuteri* KUB-AC5 (AC5), isolated from the chicken gut, confers antimicrobial and immunobiotic effects against some human pathogens. However, the antibacterial and immune modulatory effects of AC5 on UPEC have never been explored.

**Methods:**

Here, we investigated both the direct and indirect effects of AC5 against UPEC isolates (UTI89, CFT073, and clinical MDR UPEC AT31) *in vitro*. Using a spot-on lawn, agar-well diffusion, and competitive growth assays, we found that viable AC5 cells and cell-free components of this probiotic significantly reduced the UPEC growth of all strains tested. The human bladder epithelial cell line UM-UC-3 was used to assess the adhesion and pathogen-attachment inhibition properties of AC5 on UPEC.

**Results and discussion:**

Our data showed that AC5 can attach to UM-UC-3 and decrease UPEC attachment in a dose-dependent manner. Pretreatment of UPEC-infected murine macrophage RAW264.7 cells with viable AC5 (multiplicity of infection, MOI = 1) for 24 hours enhanced macrophage-killing activity and increased proinflammatory (*Nos2*, *Il6*, and *Tnfa*) and anti-inflammatory (*Il10*) gene expression. These findings indicate the gut-derived AC5 probiotic could be a potential urogenital probiotic against MDR UTI.

## Introduction

Urinary tract infections (UTIs) are some of the most common bacterial infections that harm the global economy and quality of human life (Wagenlehner et al., 2016; Medina and Castillo-Pino, 2019; Ozturk and Murt, 2020). A recent systematic review demonstrated that there are approximately 400 million UTI cases per year worldwide (Yang et al., 2022). UTIs are a significant cause of morbidity in females of all ages, young boys, and older men (Flores-Mireles et al., 2015). It has been estimated that more than 50% of women have experienced UTIs at least once in their lifetime (Griebling, 2005; Salvatore et al., 2011). UTIs are differentiated into lower UTIs (cystitis) and upper UTIs (pyelonephritis), with outcomes depending on the host and microbial factors (Bunduki et al., 2021). The morbidity and mortality rates of UTIs have increased in the last three decades, along with the rise of multidrug resistance (MDR) uropathogens. Several significant complications can result from untreated or severe forms of UTIs, including urosepsis, renal scarring in young children, and preterm birth in pregnancy (Radu et al., 2023; Su et al., 2024).

UPEC is a Gram-negative rod bacterium belonging to Enterobacteriaceae family in the phylum Pseudomonadota. The pathogenesis of UPEC UTIs has been previously reviewed (Flores-Mireles et al., 2015; Dickson et al., 2024). UPEC is a commensal residing in the gut. These strains colonize the host urethra and ascend to the bladder. Once inside the host bladder, UPEC attaches to and invades the bladder urothelium using its virulence factors, such as type I-P fimbriae (Martinez and Hultgren, 2002). Adhesion and invasion are two crucial steps for UPEC to establish an intracellular niche in host bladder urothelium critical for UPEC pathogenesis. Some UPEC strains evade the host immune response by hiding inside the bladder urothelium or undergoing morphological changes. Immune responses to UPEC in the bladder, especially those driven by neutrophils and UPEC virulence factors, cause bladder inflammation, known as cystitis. UPEC secretes cytotoxins and proteases to damage bladder epithelium and can ascend to the kidneys, resulting in pyelonephritis. Once UPEC crosses the tubular epithelial cell barrier in the kidneys, septicemia can occur.

Macrophages are innate immune cells crucial during UPEC infection (Schiwon et al., 2014; Carey et al., 2016). Macrophages perform intracellular UPEC killing by secretion of antimicrobial peptides and the production of lytic enzymes and reactive oxygen and nitrogen species (Fang, 2004; Weiss and Schaible, 2015). A recent study showed that macrophage populations in the mouse bladder are heterogeneous (Lacerda Mariano et al., 2020). Both pro-inflammatory and anti-inflammatory macrophage profiles in mice were reported. Moreover, macrophages play a role in controlling the migration of neutrophils, another important innate immune cell, into UPEC-infected urothelium (Schiwon et al., 2014).

*Limosilactobacillus* spp., formerly named *Lactobacillus*, are Gram-positive lactic acid bacteria (LAB) that exert both antimicrobial and immune modulatory effects against several human pathogens, including UPEC (Cadieux et al., 2009; Delley et al., 2015; Reid, 2023). Compounds secreted by urogenital probiotic strains *Lactobacillus rhamnosus* GR-1 and *L. reuteri* RC-14 inhibit UPEC adhesion and growth by downregulating type 1-P fimbriae expression (Cadieux et al., 2009). The administration of probiotic *Lactobacillus* vaginally or orally may prevent UTI in women (Falagas et al., 2006; Hanson et al., 2016; Akgul and Karakan, 2018). Several strains of commercial probiotic *Lactobacillus* spp. inhibit UPEC growth through environmental acidification and decreasing UPEC adhesion to human bladder urothelium (Delley et al., 2015). *L. acidophilus* (ATCC 4356) and *L. casei* (ATCC 393) exhibit antibacterial and antibiofilm activities against UPEC strains (Soltani et al., 2022). The important role of microbiota and its interventions by probiotics in UTIs has been recently reviewed (Reid, 2023).

*Limosilactobacillus reuteri* (*L. reuteri*) is a well-investigated probiotic bacterium that can colonize several sites of the human body (Mu et al., 2018; Forsgard et al., 2023; Luo et al., 2023)*. L. reuteri* is one of the common vertebrate symbionts that have evolved ecological and evolutionary strategies as a gut microbe and play a significant role in the host’s health (Walter et al., 2011). Mechanistically, *L. reuteri* provides several host benefits, especially in infectious and immune-related diseases (Park et al., 2023). First, *L. reuteri* inhibits the growth of pathogens via the secretion of antimicrobial molecules, organic acids, ethanol, antimicrobial peptides, and reuterin. Second, *L. reuteri* can modulate the host immune system in several ways, including (1) enhancing the phagocytic activity of macrophages, (2) increasing proinflammatory cytokine production, (3) promoting regulatory T-cell development and function, and (4) providing an anti-inflammatory effect. Third, *L. reuteri* improves gut health by strengthening the intestinal barrier. Applications of *L. reuteri* in the prevention and treatment of several human immune-related diseases have been recently reviewed by Luo Z. et al (Luo et al., 2023). However, the strain-specific and immunobiotic effects of *L. reuteri* in UPEC infection have been relatively under-investigated. Here, we studied the *in vitro* antimicrobial and immunomodulatory effects of *L. reuteri* strain KUB-AC5, isolated from chicken intestine, against UPEC isolates.

## Materials and methods

### Ethical approvals

The clinical UPEC strain (AT31) was isolated from the urine of a ninety-year-old male patient with post-operative UTI due to prostate cancer admitted to Maharaj Nakorn Chiang Mai Hospital (MNCMH) in 2020. The retrospective study on his medical record was approved by the Research Ethics Committee, Faculty of Medicine, Chiang Mai University (Approval No. 097/2567). This study was approved by the institutional biosafety committee, Faculty of Medicine, Chiang Mai University, Thailand (Approval No. CMUIBC02012/2565).

### Bacterial strains and culture conditions

All bacterial strains in this study are shown in **Supplementary Table 1**. The UPEC strains were grown by shaking at 37°C in Luria-Bertani (LB) broth (Difco, Sparks, MD, US) for 16–18 h. For UTI89, 0.05 mg/mL kanamycin (AppliChem, Germany) was added to culture media as a selective antibiotic. Chicken intestine-originated probiotic *Limosilactobacillus reuteri* strain KUB-AC5 (Nitisinprasert et al., 2000; Nakphaichit et al., 2019) was cultured statically (microaerophilic) without shaking at 37°C in De Man, Rogosa and Sharpe (MRS) broth (Difco, Sparks, MD, US) for 16–18 h unless stated otherwise. All bacterial cultures were performed at the ambient atmospheric gas conditions.

### PCR for UPEC virulence factor detection in clinical isolate UPEC strain AT31

We detected the presence of four UPEC-specific genes (*c3509*, *c3686*, *chuA*, and *uidA*) with three significant virulence genes (*fimH*, *Sfa*, *iroN*) by PCR using primer pairs listed in **Supplementary Table 2**. UPEC strains UTI89 and CFT073 were used as positive controls, while *Salmonella enterica* serovar Typhimurium strain IR715 was used as a negative control. The PCR condition was 95°C for 30 s; 35 cycles each of 95°C for 7 s, 60°C for 12 s, 75°C for 12 s; and 72°C for 30 s.

### Calculating the average nucleotide identity matrix

Bacterial whole genomic DNA of AT31 was extracted by Dneasy Ultraclean Microbial kit (Qiagen, Germany) and subsequently sequenced via NovaSeq 6000 Sequencing System (Illumina, San Diego, CA, US). Then, genome sequences of 15 species closely related to the UPEC were sourced from the NCBI database in July 2023. Sequence similarity between any two genomes was computed using pyani v0.2.12, a Python package that calculates a measure of nucleotide-level genomic similarity between microbial genomes ANI based on the BLAST (Basic Local Alignment Search Tool) method (ANIb). In the context of ANIb, BLAST is used to align genome fragments between pairs of genomes and calculate the percentage of nucleotide identity for these alignments. The pyani tool breaks each genome into smaller fragments and aligns these fragments between pairs of genomes. For each alignment, it calculates the percentage identity. The ANI was then computed as the mean percentage identity of all these fragment alignments, providing a measure of how similar the two genomes are at the nucleotide level. The similarity results were subsequently visualized using ggplot2 v3.4.2 Elegant Graphics for Data Analysis (Springer-Verlag New York) retrieved from https://ggplot2.tidyverse.org, a data visualization package for the R programming language. A triangle heatmap was used to present the ANI results. In a triangle heatmap, each cell represents the ANI value between the pair of genomes, and color intensity indicates the level of similarity. Clusters of closely related species were identified based on color patterns. The genome accession numbers are shown in **Supplementary Table 5**.

### Construction of phylogenetic tree of AT31 based on core genome MLST

Genome sequences of *E. coli* were used to construct a core genome (cg) MLST-based phylogenetic tree for AT31. The core genes of these strains were identified using panX v1.6.0 (Ding et al., 2018), followed by the creation of a multiple sequence alignment. The multiple sequence alignment, initially generated in the aln file format, was converted to the phylip file format using trimal v1.2 (Capella-Gutierrez et al., 2009). The resulting multiple sequence alignment was then employed for the reconstruction of a phylogenetic tree through 100 bootstrapping iterations using RAxML v8 (Stamatakis, 2014). Then, the phylogenetic tree was visualized using iTOL v6.8 (Letunic and Bork, 2021).

### Antibiotic susceptibility test for the clinical isolate UPEC AT31

Antibiotic susceptibility testing was conducted via the Kirby-Bauer method and as previously described (Buddhasiri et al., 2023). Briefly, the bacterial colonies were resuspended in 3 mL PBS until the turbidity of the suspension reached 0.5 McFarland. The suspension was then swabbed and streaked evenly over the surface of Mueller-Hinton (MH) agar (approximately 4 mm depth). Then, antimicrobial-impregnated disks (Oxoid, UK) were placed on the agar. The clear zone diameter was measured after 18 h of incubation at 37°C at the ambient gas condition. Antibiotic susceptibility was interpreted following the 2020 Clinical & Laboratory Standards Institute (CLSI) guideline. The list of antibiotics used and the results are shown in **Supplementary Table 3**.

### Spot-on-lawn and agar diffusion assay

To determine the direct anti-UPEC effect of viable cells and cell-free components of AC5, spot-on-lawn and agar diffusion assays were performed, respectively (Lima et al., 2007; Buddhasiri et al., 2021). A single colony of AC5 was inoculated into 5 mL MRS broth and incubated at 37°C for 16-18 h without shaking. 20 μL of AC5 overnight culture was directly spotted on the surface of MRS agar and incubated at 37°C for 16-18 h. Then, 20 mL of LB broth containing 0.75% agar mixed with UPEC overnight culture (100:1 ratio) was poured onto the AC5-spotted MRS agar and incubated at 37°C for 16 hr. The diameter of the inhibition zone was measured (> 1 mm considered positive).

For the agar-well diffusion assay, AC5 cell-free supernatant (CFS) was prepared by centrifugation of 5 mL AC5 overnight culture at 4,000 rounds per minute (rpm), 4 °C for 10 min. Then, the supernatant was collected and filtered through 0.45 μm-pore size filter paper. 60 μL of the filtrate was filled into an agar well with a diameter of 6 mm made by 0.75% LB agar mixed with UPEC overnight culture (100:1 ratio). 60 μL of 0.05 mg/mL nalidixic acid (AppliChem, Germany) was used as a positive control. The diameter of the inhibition zone was measured (> 1 mm considered positive).

### Bacterial co-culture assay

A bacterial co-culture assay was performed by competitive growth of UPEC and AC5 (1:1) in the co-culture media prepared from a 1:1 mixture of MRS and MH broth (Difco, Sparks, MD, US) at 37°C, statically without shaking. Approximately 5 x 10^4^ cfu/mL of AC5 and UPEC strains were equally added. Then, 500 μL of the bacterial solution was harvested in the indicated time points, and colony-forming unit (cfu)/mL was enumerated by a serial-ten-fold plating technique. Each strain’s single growth (monoculture) in the co-culture media was done separately.

### Cell culture for RAW264.7 and UM-UC-3

The murine macrophage cell line RAW264.7 (ATCC TIB-71), and human bladder epithelial cell line UM-UC-3 (ATCC CRL-1749) were cultured in a T75 flask until the confluence reached 60-80% at 37°C with 5% CO_2_ in a humidified incubator. Complete growth media for RAW264.7 contained Dulbecco’s Modified Eagle’s Medium-high glucose (DMEM; 4 mM L-glutamine, 4500 mg/L glucose with sodium pyruvate, Cytiva, Utah, US), 10% fetal bovine serum (FBS) and 1% Penicillin/Streptomycin (Cytiva, Utah, US). Eagle’s Minimum Essential Medium (EMEM; 2 mM L-glutamine with sodium pyruvate, ATCC, Manassas, VA, US) with 10% FBS and 1% Penicillin/Streptomycin was used as a complete growth medium for UM-UC-3.

### Probiotic adhesion and adhesion-inhibition assays on UM-UC-3

For adhesion assays, human bladder urothelial UM-UC-3 cells were seeded into a 24-well plate at a density of 5x10^5^ cells per well and grown at 37°C with 5% CO_2_ for 24 h. Then, the culture medium was replaced by serum- and antibiotic-free EMEM and incubated at 37°C with 5% CO_2_ for another 24 h. Each well was inoculated with 100 μL of different doses (10^6^, 10^7^, 10^8^, and 10^9^ cfu/mL) of probiotic AC5 or ABU83972 (equal to MOI = 1, 10, 100, 1000, respectively). Cells were washed with 0.5 mL DPBS three times after 24 h of incubation. 0.5 mL of 0.25% trypsin was added into each well and incubated for 10 minutes until cells detached from well surfaces. The cell suspension was collected for enumeration of the bacteria that adhered to UM-UC-3 (cfu/mL) by a standard 10-fold diluting technique.

For the adhesion-inhibition assay, UM-UC-3 cells were prepared as stated above. The inocula of UPEC strains (UTI89, CFT073, and AT31) were grown in LB broth supplemented with 0.3 M NaCl at 37°C with shaking for 2 h until the OD600 reached 0.6-0.9 (Winter et al., 2009). Then, cells were infected with UPEC strains (MOI = 1) and incubated for 1 h. The infected cells were washed three times with DPBS. 0.5 mL of 0.25% trypsin was added into each well and incubated for 10 minutes until cells lifted off. The cell suspension was collected for enumeration of the bacteria (both AC5 and UPEC) that adhered to UM-UC-3 (cfu/mL) by a standard 10-fold diluting technique.

### Gram staining of the adhered AC5 on UM-UC-3

To observe the adhesive property of AC5 on UM-UC-3, the UM-UC-3 cells were grown on a 6-well-plate (10^6^ cells/well) and washed twice with sterile PBS before adding 3 mL of RPMI1640 to each well then incubated at 37°C with 5% CO_2_ for 24 hours. Later, 10^8^ cfu of AC5 were added into each well and incubated at 37°C with 5% CO_2_ for another day (24 hours). Cells were washed with PBS three times to eliminate the non-adhered bacteria, fixed with 3 mL methanol, and incubated at room temperature for 10 minutes. After removing methanol, cells were stained with the Gram stain kit (BD Bioscience, USA) and air dried. The picture was taken under an oil-immersion objective lens using Olympus IX71, DP73 (Olympus, Tokyo, Japan).

### Gentamicin protection assay for invasion and macrophage phagocytic killing

UM-UC-3 and RAW264.7 cells were seeded into 24-well plates (approximately 5 x 10^5^ cells per well). The gentamicin protection assay was performed as previously described with slight modification (Winter et al., 2009). Briefly, each well containing UM-UC-3 and RAW264.7 was pretreated with viable probiotic AC5 (MOI = 1) and incubated at 37°C, 5% CO_2_ for 24 h. Then, the pretreated wells were washed three times with sterile DPBS and subsequently infected with UPEC strains (MOI = 1) for 1 h. Each well was washed twice with DPBS, and 0.5 mL media containing 100 μg/mL of gentamicin sulfate (AppliChem, Germany) was added. The plate was incubated at 37°C for 90 min to kill the extracellular UPEC population. Cells were lysed with 1% Triton-X-100 (AppliChem, Germany) in PBS to collect suspensions containing previously intracellular UPEC.

### Detection of immune-related gene expressions by quantitative polymerase chain reaction

RAW264.7 cells were seeded into a 6-well-plate at a density of approximately 1 x 10^6^ cells per well and incubated at 37°C with 5% CO_2_ for 24 h. Then, the media were replaced with serum and antibiotic-free DMEM high-glucose for 24 h. Cells were pretreated with viable AC5 (MOI = 1) for 24 h before being infected with UPEC strains (MOI = 1) for 1 h. Then, 1 mL TRIzol reagent (Invitrogen, Carlsbad, CA, US) was added into each well to collect the RNA. The RNA extraction was performed following the manufacturer’s protocol. RevertAid First Strand cDNA reagents (Thermo Fisher Scientific, Lithuania) were used for cDNA generation. The qPCR for gene expression was performed by using the SensiFAST SYBR Lo-ROX Kit (Meridian Bioscience, Memphis, Tennessee, US) and ViiA 7 Real-Time PCR system (Applied Biosystems). The data was then normalized by a housekeeping gene *Gapdh* and fold changes of gene expression were calculated by the comparative Ct method as previously described (Sarichai et al., 2020). Primers used for the qPCR are listed in **Supplementary Table 4**.

## Results

### The clinically isolated AT31 is multi-drug resistant and closely related to the pyelonephritic CFT073 and asymptomatic bacteriuria ABU83972 UPEC strains

**Figure 1:**
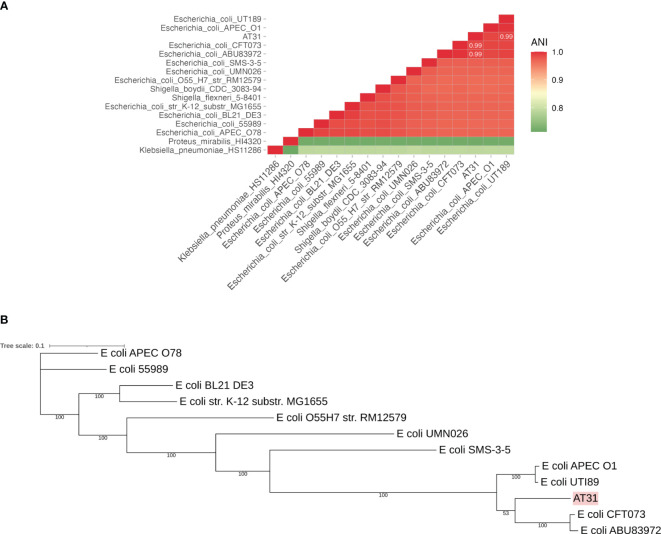
Average nucleotide identity (ANI) matrix **(A)** and phylogenetic tree of clinical isolate uropathogenic *E. coli* AT31 and other related species **(B)**. AT31 is a UPEC strain isolated from the urine of a ninety-year-old male patient admitted to Maharaj Nakorn Chiang Mai Hospital (MNCMH) in 2020, and it is closely related to UPEC CFT073 and ABU83972. ANI was calculated by the sequence similarity between any of the two genomes. The phylogenetic tree was constructed based on the core genome (cg) MLST.

AT31 was isolated from a urine culture of a 90-year-old man diagnosed with prostate cancer and bacterial UTI after the prostatectomy admitted to the MNCMH in 2020. PCR showed the presence of UPEC-specific genes (*c3509*, *c3686*, *chuA*, and *uidA*) and virulence genes (*fimH*, *Sfa*, and *iroN*) in AT31 (**Supplementary Figures S1A, B**, respectively). Bacterial genomic DNA was extracted and subsequently sequenced via the NovaSeq 6000 Sequencing System (Illumina). The calculation of the average nucleotide identity (ANI) matrix and a core genome MLST-based phylogenetic tree indicated AT31 is a UPEC closely related to the pyelonephritic CFT073 and asymptomatic bacteriuria ABU83972 strains (**Figures 1A, B**, respectively). Moreover, the MDR phenotype of AT31 was investigated with the disc assay. Our data revealed that AT31 is MDR and resistant to five antibacterial agents (**Supplementary Table 3**).

### Probiotic *L. reuteri* KUB-AC5 directly inhibited the growth of UPEC strains

**Figure 2:**
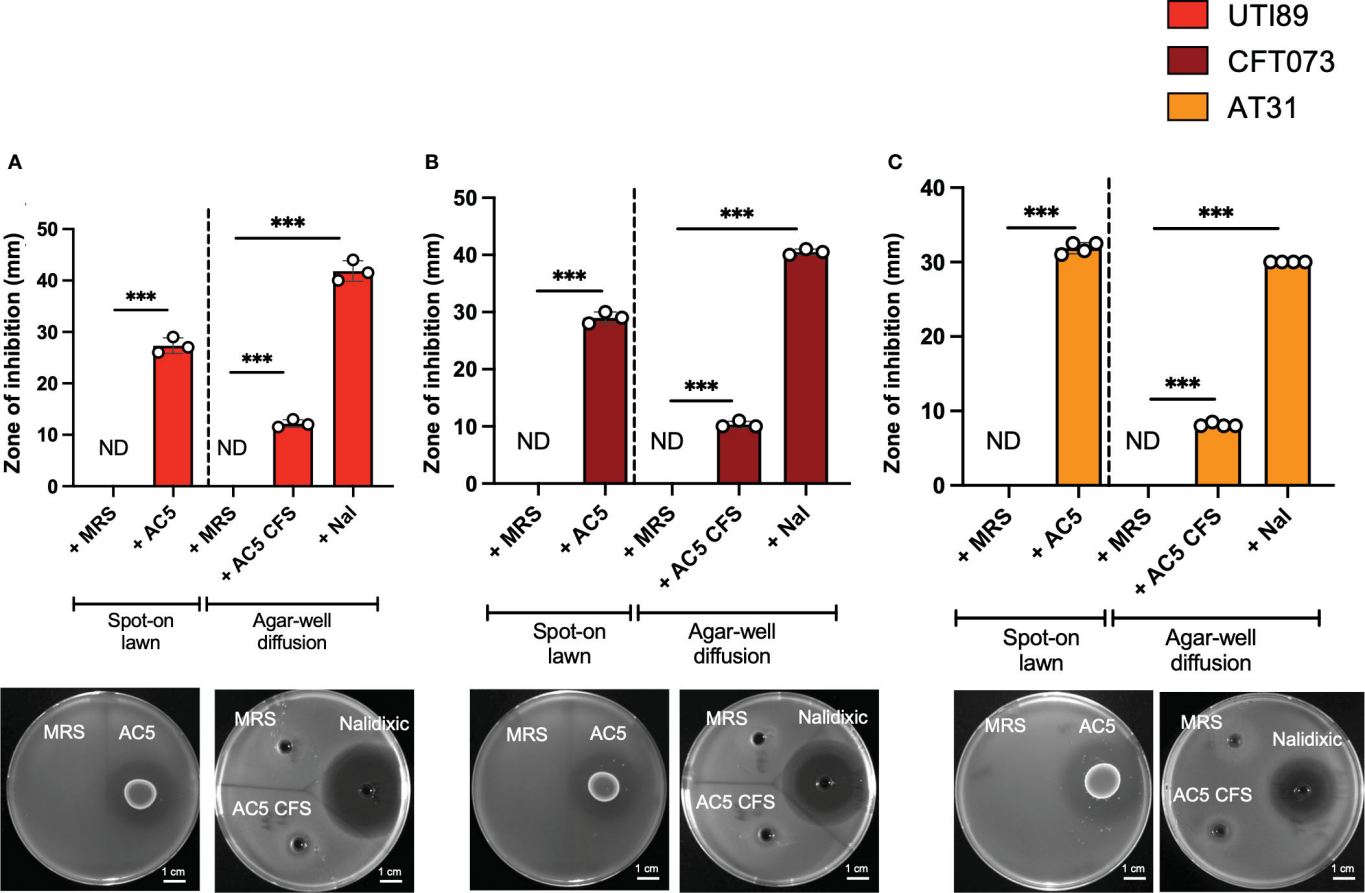
Viable and cell-free probiotic *L. reuteri* KUB-AC5 directly inhibit the growth of UPEC strains. Live AC5 and AC5 cell-free supernatant (CFS) were used in spot-on lawn and agar-well diffusion assays, respectively, against UPEC strains UTI89 **(A)**, CFT073 **(B)**, and clinical AT31 **(C)**. An anti-UPEC effect of AC5 was observed based on the presence of a clear zone (> 1 mm diameter in both assays). Nal; nalidixic acid as a positive control. MRS; De Man, Rogosa and Sharpe (MRS) broth as a negative control. ND, non-detectable. Bars represent geometric means +/- the standard deviation of at least triplicates. *** indicate *P*-value < 0.001.

The direct anti-UPEC effects of live and cell-free supernatant (CFS) forms of AC5 were investigated by spot-on lawn and agar-well diffusion assays, respectively. Clear zones surrounding the spots of viable AC5 and AC5 CFS-filled wells were observed in all three UPEC strains (UTI89, CFT07, and AT31) (**Figures 2A–C**, respectively).

**Figure 3:**
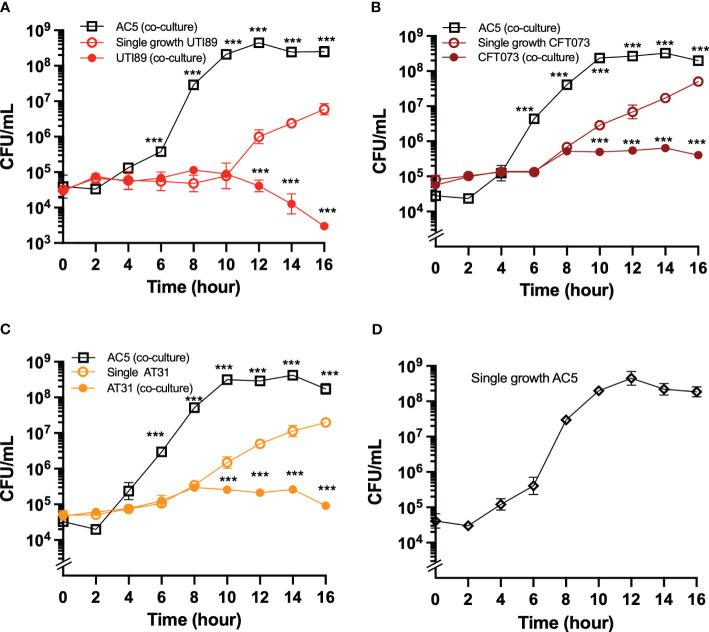
The temporal kinetics of anti-UPEC properties of probiotic *L. reuteri* KUB-AC5 in co-culture. UPEC strains UTI89 **(A)**, CFT073 **(B)**, and clinical AT31 **(C)** were either co-cultured (competitive growth) with an equal amount of AC5 (1:1 ratio) or monocultured (single growth). The number of both AC5 and UPEC were determined as CFU/mL as the indicated timepoint. The single growth of AC5 (i.e., no other bacteria in culture) in the same co-culture media was also illustrated **(D)**. All data is represented in geometric means +/- the standard deviation of at least triplicates. *** indicate *P*-value < 0.001.

Next, we investigated the temporal kinetic study of the anti-UPEC effect of viable AC5 on UPEC strains by using a co-culture assay. A 1:1 ratio inoculum between AC5 and UPEC strains was incubated statically at 37°C, and the numbers of bacteria were determined at the indicated timepoint (**Figure 3**). We found that the growth of all three UPEC strains (UTI89, CFT073, and AT31) was significantly inhibited in the presence of AC5 (co-culture) compared to monoculture (**Figures 3A–C**, respectively). The single growth of AC5 in co-culture media was also determined (**Figure 3D**).

### High dose AC5 reduced UPEC adhesion and invasion to human bladder urothelium

**Figure 4:**
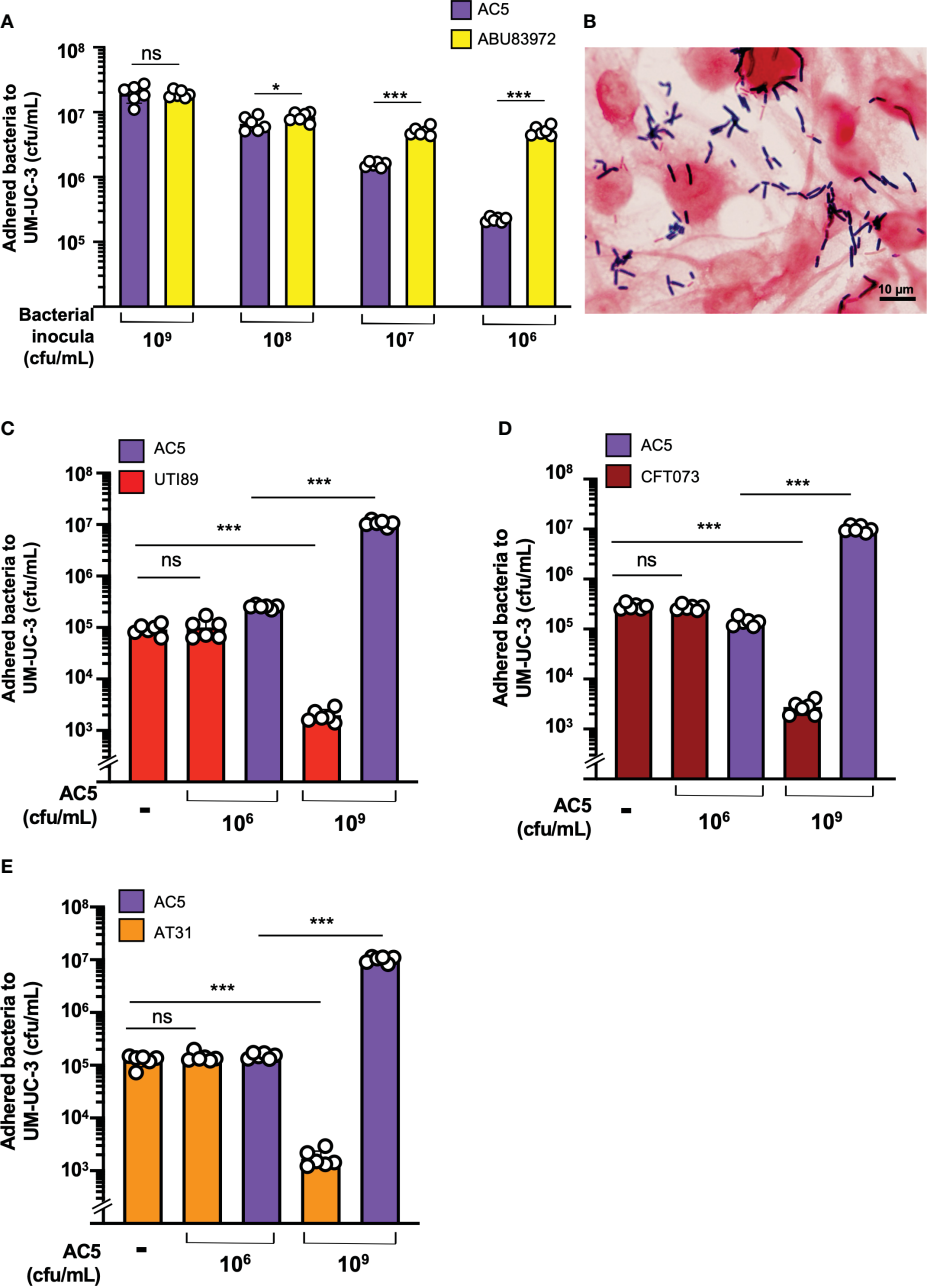
Dose-dependent effect of probiotic *L. reuteri* KUB-AC5 on the adhesion of UPEC strains to human bladder urothelium (UM-UC-3). At high-dose inoculum (10^9^ cfu/mL), AC5 can adhere to UM-UC-3, not differing from the urogenital probiotic strain ABU83972 **(A)**. A representative picture of Gram’s staining demonstrates the adhesion of AC5 (purple) on human bladder urothelium (red) **(B)**. Pretreatment with high dose AC5 (10^9^ cfu/mL) significantly reduced the numbers of UPEC strains: UTI89 **(C)**, CFT073 **(D)**, and AT31 **(E)**. Bars represent geometric means +/- the standard deviation of at least triplicates. *, *** indicate *P*-value < 0.05 and 0.001, respectively. ns; non-statistically significant difference. – indicates absence.

**Figure 5:**
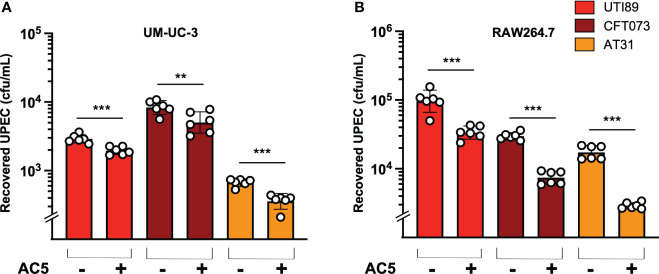
Probiotic *L. reuteri* KUB-AC5 reduced intracellular survival of UPEC strains in host cells. Pretreatment with AC5 (MOI = 1) for 24 h significantly decreased the recovered intracellular numbers (cfu/mL) of all UPEC strains (UT189, CFT073, and AT31) from human urothelial UM-UC-3 cells **(A)** and murine macrophage RAW264.7 cells **(B)**. Bars represent geometric means +/- the standard deviation of at least triplicates. **, *** indicate *P*-value < 0.01 and 0.001, respectively. – and + indicate absence and presence, respectively.

The dose-dependent effect of AC5 on its adhesion to human bladder urothelial cells (UM-UC-3) was determined. We found that a high dose of AC5 (10^9^ CFU/mL) adhered to human bladder urothelium, similar to the asymptomatic bacteriuria strain of *E. coli* ABU83972. However, low doses of AC5 (10^8,^10^7^, and 10^6^ cfu/mL) showed reduced adhesion to UM-UC-3 compared to that of ABU83972 (**Figure 4A**). After three PBS washes, Gram staining of high-dose AC5 (10^9^ cfu/mL) and UM-UC-3 revealed purple rod-shaped bacilli attached to the red UM-UC-3 (**Figure 4B**). Next, we investigated whether AC5 could reduce the adhesion of different UPEC strains on UM-UC-3 by the adhesion-inhibition assay. Our data showed that only high dose AC5 could significantly reduce the numbers of adhered UTI89, CFT073, and AT31 on UM-UC-3 (**Figures 4C–E**, respectively). Moreover, we found that AC5 can significantly reduce the number of intracellular UPEC in UM-UC-3 cells (**Figure 5A**).

### Pretreatment with AC5 enhances phagocytic killing activity and immune-related gene expression in murine macrophages

**Figure 6:**
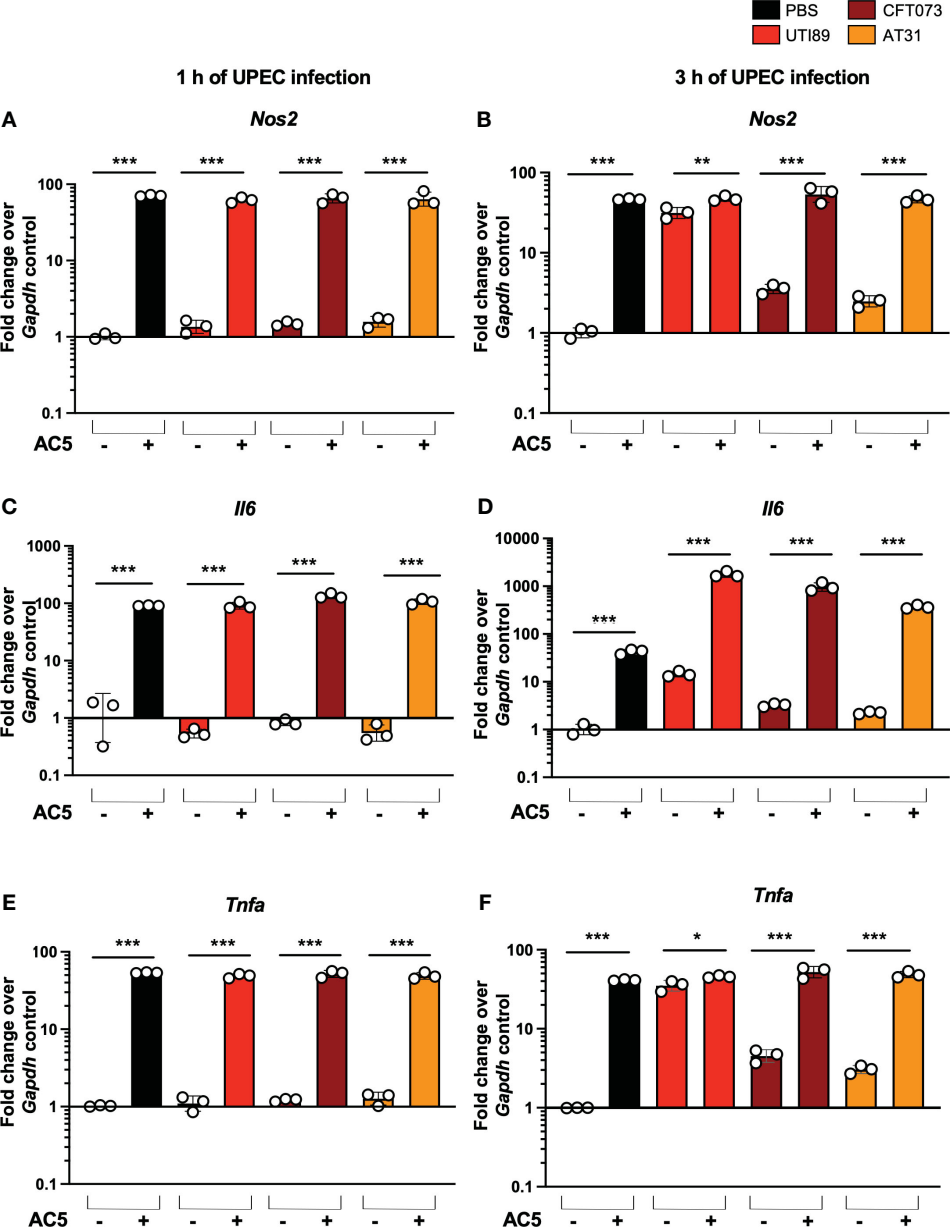
Immune modulatory effect of viable probiotic *L. reuteri* KUB-AC5 on murine macrophage RAW264.7. Pre-treatment with AC5 (MOI =1) increased expression of the proinflammatory genes *Nos2*, *Il6*, and *Tnfa* in RAW264.7 at 1 h after UPEC infection (MOI = 1) (**A, C, E**, respectively). Pre-treatment with AC5 also significantly enhanced *Nos2*, *Il6*, and *Tnfa* mRNA expression at 3 h after UPEC infection (**B, D, F**, respectively). Bars represent geometric means +/- the standard deviation of at least triplicates. *, **, *** indicate *P*-value < 0.05, 0.01, and 0.001, respectively. – and + indicate absence and presence, respectively.

**Figure 7:**
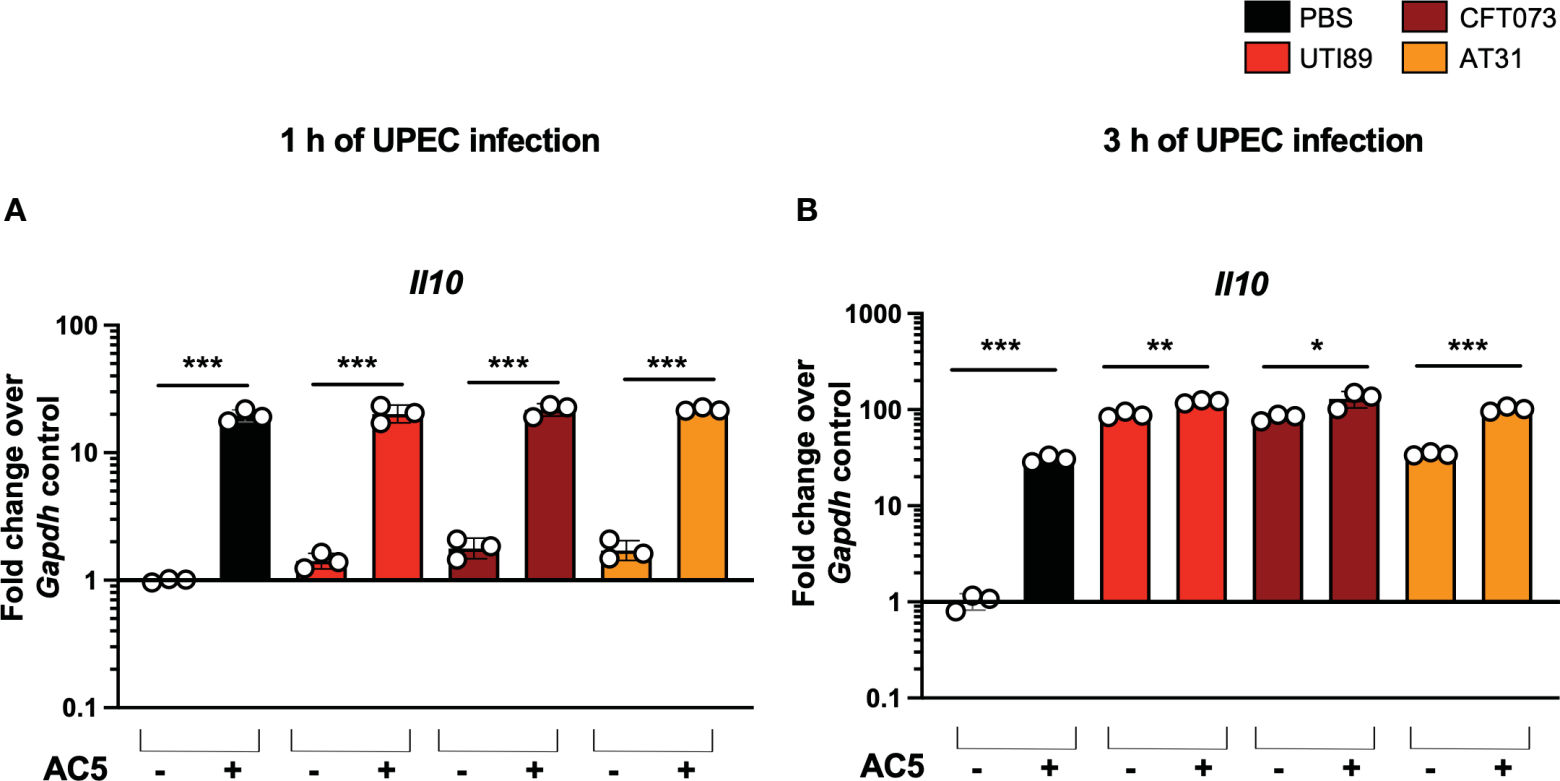
Viable probiotic *L. reuteri* KUB-AC5 induced anti-inflammatory cytokine gene *Il10* expression in murine macrophages. Pre-treatment with AC5 (MOI = 1) increased *Il10* expression in RAW264.7 at 1 and 3 after UPEC infection (MOI = 1) (**A, B**, respectively). *, **, *** indicate P-value < 0.05, 0.01, and 0.001, respectively. – and + indicate absence and presence, respectively.

By using a gentamicin protection assay, we found that pretreatment with viable AC5 for 24 h significantly increased the phagocytic activity of murine macrophage RAW264.7 cells infected with UTI89, CFT073, and AT31 (**Figure 5B**). A reduction in all UPEC strain numbers recovered from the AC5-pretreated RAW264.7 cells indicated an increase in macrophage killing activity compared to the untreated cells. Next, we determined the immune modulatory effect of AC5 on macrophages by measuring the gene expression of proinflammatory proteins via qPCR (**Figure 6**). Our data showed that pretreatment with AC5 increased expression of *Nos2*, *Il6*, and *Tnfa* encoding for inducible nitric oxide synthase (iNOS), interleukin (IL)-6, and tumor necrosis factor (TNF) alpha, respectively, at 1 and 3 h after UPEC infection. Interestingly, pretreatment with AC5 also increased immunosuppressive cytokine *Il-10* gene expression at 1 and 3 h after UPEC infection (**Figure 7**).

## Discussion

Probiotics have been considered promising alternative treatments for several human bacterial infections, including UPEC UTI (Falagas et al., 2006; Hanson et al., 2016; Akgul and Karakan, 2018). Direct bacterial inhibitory activities, such as antimicrobial substance secretion, strong acidic pH environment, and competition for nutrients or space in the host niche, are known as important mechanisms of probiotics against pathogens (Sassone-Corsi and Raffatellu, 2015). Indirect bacterial inhibitory actions, including immunomodulatory effects via the activation of host immune cells by microbial ligands or metabolites, is also a significant mechanism of probiotics against pathogens.

Lactic acid bacteria (LAB), such as the Gram-positive rod bacterium in the genus *Limosilactobacillus*, previously named *Lactobacillus*, is one of the extensively studied probiotics (Di Cerbo et al., 2016). However, there are differences in their activities when it comes to the applications. Mostly from the strain-specific effect of probiotics and their host ranges (Liu et al., 2023; Peng et al., 2023; Hmar et al., 2024). AC5 was isolated from the chicken intestine and conferred the anti-bacterial effect against several human pathogens, including *E. coli* and *Salmonella* (Nitisinprasert et al., 2000; Buddhasiri et al., 2021). However, the anti-UPEC effect of AC5 has never been investigated.

Here, the direct and indirect effects of AC5 against three UPEC strains (UTI89, CFT073, and AT31) were investigated. The UPEC strain UTI89, a cystitis strain originally isolated from a girl with a bladder infection (Mulvey et al., 2001). The UPEC strain CFT073 is a pyelonephritogenic strain isolated from blood samples of a patient with pyelonephritis (Mobley et al., 1990). The UPEC strain AT31 was isolated from a complicated UTI case admitted to the MNCMH and characterized by PCR and WGS in this study. The antibiotic susceptibility profiling of AT31 demonstrates its MDR phenotype by resistant to five antibiotics. The AT31 genome contains common virulence genes *fimH*, *Sfa*, and *iroN* and is phylogenetically related *to E. coli* CFT073 and ABU83972 in the phylogenetic group B2.

Previous studies showed that the commensal and probiotic *Limosilactobacillus* produces various organic and non-organic compounds that feature antimicrobial activity on uropathogens including UPEC (Servin, 2004). Nonetheless, the antimicrobial effectiveness of probiotics depends on specific strains, pathogen properties, and the presence of other compounds in the environment. Several strains of *Limosilactobacillus* spp. exhibit both lactic acid-dependent and independent antimicrobial activities against *Salmonella enterica* Typhimurium (Fayol-Messaoudi et al., 2005). For instance, the inhibitory effect of *Limosilactobacillus* against UTI89 and CFT073 *in vitro* is largely based on high levels of acid production (Delley et al., 2015). Moreover, the acid-independent antimicrobial effect of AC5 was also previously shown (Nitisinprasert et al., 2000). AC5 conferred a bacteriocin-like activity that is not based on a bacteriocin by producing antimicrobial peptides that inhibit *E. coli* and *Salmonella* growth, and this activity was attenuated in low pH conditions.

The study by our group already showed that AC5 inhibits the growth of *Salmonella enterica* serovar Typhimurium and decreases gut inflammation in mice (Buddhasiri et al., 2021). Here, our data illustrates that live AC5 and its cell-free component also directly inhibit the growth of UPEC strains. The direct inhibitory effect of AC5 on several Enterobacteriaceae was previously shown (Sobanbua et al., 2020). AC5 produces a heat-stable non-bacteriocin-like antimicrobial peptide KAC5 to inhibit the spectrum of both G+ and G- bacteria but not LAB, including *L. reuteri*. The antimicrobial effect of KAC5 might come from the combination of acids with other unknown antimicrobial substances. Interestingly, AC5 does not convert glycerol into reuterin (3-hydroxypropionaldehyde), a potent, broad-spectrum antimicrobial substance against *E. coli* and *Salmonella*, that is commonly found in other *L. reuteri* (Schaefer et al., 2010).

Here, we used cell culture models of human urothelium UM-UC-3 and murine macrophage RAW264.7 cells to investigate the anti-bacterial and immunomodulatory effects of AC5. The dose-dependent effect of AC5 in the adhesion and inhibition of UPEC strains to human urothelium is shown in this study. A high dose of AC5 is needed to prevent all UPEC adhesions on human bladder urothelium in comparison with the prototypic urogenital strain of *E. coli* probiotic ABU83972. This indicated the broad spectrum of AC5 in the direct anti-UPEC adhesion to human bladder epithelium. Possible mechanisms of the anti-adhesion from urogenital probiotic *Limosilactobacillus* were previously reported (Cadieux et al., 2009).

Cadieux P. et al. showed that lactic acid and cell-free supernatant of *Lactobacillus rhamnosus* GR-1 and *L. reuteri* RC-14 inhibit the growth of UPEC C1212 by downregulating the expression of UPEC type 1-P fimbriae in a dose- and pH-dependent manner. The reduction in type 1-P fimbriae expression, critical virulence factors for urothelium adhesion, resulted in attenuated human bladder urothelium cell adhesion. This suggested that *Lactobacillus* metabolites could reduce the expression of both mannose-sensitive (type 1) and resistant (P) fimbriae. UPEC type 1 fimbriae tip protein FimH binds to mannose on urothelial uroplakin while the type P fimbriae binds to host glycosphingolipids (Tchesnokova et al., 2011; Legros et al., 2019; Whelan et al., 2023). The displacement of the adhering UPEC on UM-UC-3 by the strains of commercial probiotic *Lactobacillus* was also reported (Delley et al., 2015). This suggests the possibility of using AC5 as a urogenital probiotic since it can adhere to and reduce UPEC adhesion to human bladder epithelium. However, the effect of AC5 in the reduction of UPEC fimbrial gene expressions has not been investigated in this study.

Besides the fact that UPEC can invade and replicate within bladder epithelial cells, UPEC can also survive inside the host macrophage to gain an intracellular niche within the host (Bokil et al., 2011). Macrophage is the innate immune cell that plays a crucial role in defending against intracellular bacterial infection, including UPEC, by phagocytosis and production of pro-inflammatory mediators (Bokil et al., 2011; Weiss and Schaible, 2015). Interestingly, some UPEC strains can attenuate macrophage-killing activity, and the host species differences may impact UPEC survival in the host. Our data showed that pretreatment of murine macrophage with viable AC5 can enhance the phagocytic killing activity and increase the expression of proinflammatory mediators (iNOS, IL6, and TNF-alpha) at 1 and 3 h after UPEC infection. These data are consistent with several previous reports. For instance, *L. rhamnosus* GG, *L. rhamnosus* KLSD, *L. helveticus* IMAU70129, *L.casei* IMAU60214, *L. brevis* KCTC 12777BP, *and L. plantarum* KCTC 13314BP can enhance macrophage killing activity to pathogens (Jeong et al., 2019; Nanjundaiah et al., 2020; Rocha-Ramirez et al., 2020; Hwang et al., 2023).

Interestingly, we found that AC5 can induce immunosuppressive cytokine IL-10 gene expression in RAW264.7 cells. The anti-inflammatory effect of probiotic *Lactobacillus* has been formerly reviewed (Amdekar et al., 2011). Lipopeptide of *Lactobacillus* spp. activates IL-10 production from urethral epithelium via the activation of the Toll-like receptor (TLR)-2 pathway (Rachmilewitz et al., 2004). Together, these suggest that probiotic AC5 confers immunomodulatory effects on murine macrophage for both pro-inflammatory and anti-inflammatory sides.

## Conclusion

A viable form of probiotic *Limosilactobacillus reuteri* KUB AC-5 isolated from chicken intestine is a potential candidate for being a urogenital probiotic due to its ability to (1) adhere to human bladder epithelium, (2) decrease UPEC strain adhesion and invasion of human bladder epithelium, and (3) increase macrophage killing activity via immune modulation. Nonetheless, we reported these *in vitro* findings of antibacterial and immunomodulatory effects of probiotic AC5 on a few strains of UPEC. These effects of AC5 should be further investigated *in vivo* since host factors might alter AC5’s actions in the setting of UPEC UTI pathogenesis.

## Data availability statement

The datasets presented in this study can be found in online repositories. The names of the repository/repositories and accession number(s) can be found below: https://www.ncbi.nlm.nih.gov/, sra/PRJNA1083010.

## Ethics statement

The studies involving humans were approved by Research Ethics Committee Panel 5 Faculty of Medicine, Chiang Mai University. The studies were conducted in accordance with the local legislation and institutional requirements. Written informed consent for participation was not required from the participants or the participants’ legal guardians/next of kin in accordance with the national legislation and institutional requirements. Ethical approval was not required for the studies on animals in accordance with the local legislation and institutional requirements because only commercially available established cell lines were used.

## Author contributions

AT: Conceptualization, Data curation, Formal analysis, Investigation, Methodology, Validation, Visualization, Writing – original draft, Writing – review & editing. SL: Investigation, Writing – review & editing. SB: Investigation, Writing – review & editing. CS: Investigation, Writing – review & editing. PM: Investigation, Writing – review & editing. PB: Investigation, Writing – review & editing. SW: Methodology, Writing – review & editing. PP: Formal analysis, Investigation, Methodology, Visualization, Writing – review & editing. SS: Data curation, Formal analysis, Investigation, Methodology, Visualization, Writing – review & editing. MN: Resources, Writing – review & editing. SN: Resources, Writing – review & editing. MH: Resources, Writing – review & editing. PT: Conceptualization, Data curation, Formal analysis, Funding acquisition, Methodology, Resources, Supervision, Validation, Visualization, Writing – original draft, Writing – review & editing.

## Supplementary material

Supplementary Figure 1 Polymerase chain reaction confirmation of AT31 as a uropathogenic *E. coli* strain (1.2% agarose gel). Four UPEC signature genes (*c3509*, *c3686*, *chuA*, and *uidA*) were detected in AT31 **(A)**. Three UPEC virulence genes (*fimH*, *Sfa*, and *iroN*) were detected in AT31 **(B)**.
