# Supplementary material for: Probiotic Limosilactobacillus reuteri KUB-AC5 decreases urothelial cell invasion and enhances macrophage killing of uropathogenic Escherichia coli in vitro study
Source: Front Cell Infect Microbiol. 2024 Jul 18;14:1401462. doi: 10.3389/fcimb.2024.1401462 (PMC11291381; doi:10.3389/fcimb.2024.1401462)
Supplement: Supplementary file 1 [file DataSheet_1.pdf]

Supplementary Table 1. Bacterial strains used in this study

Designation	Genotype and relevant characteristic	Reference
Uropathogenic *E. coli* (UPEC) UTI89	UTI89 *attHK022::COM-GFP* (Kan^r)	(1)
Uropathogenic *E. coli* (UPEC) CFT073	Originally isolated from the blood and urine of a woman with acute pyelonephritis, ATCC-700928	(2)
Uropathogenic *E. coli* (UPEC) AT31	MDR-UPEC strain isolated from the urine of a ninety-year-old-male patient admitted to Maharaj Nakorn Chiang Mai Hospital (MNCMH) due to a post-operative urinary tract infection in 2020	This study
Limosilactobacillus reuteri KUB-AC5	Probiotic bacteria isolated from chicken intestine	(3)
Salmonella enterica Typhimurium IR715	Nalidixic acid resistance derivative of ATCC 14028	(4)

Supplementary Table 2. PCR primer list used in this study

Species	Gene	Sequence (5’->3’)	Product size (bp)	Reference
E. coli	*c3509*	ACAATCCGCCACCATCCAG	208	(5)
		CTCTCCACCGGAGAGTGTT		
	c3686	TTGCACCAACAACGTCTACC	259	
		TCTGCGTCTTCTACCATCAC		
	chuA	GCTACCGCGATAACTGTCAT	221	
		TGGAGAACCGTTCCACTCTA		
	uidA	CGCCGATGCAGATATTCGTA	259	
		CTGCCAGTTCAGTTCRTTGT		
	fimH	TGCAGAACGGATAAGCCGTGG	506	(6)
		GCAGTCACCTGCCCTCCGGTA		
	Sfa	CTCCGGAGAACTGGGTGCATCTTAC	410	(7)
		CGGAGGAGTAATTACAAACCTGGCA		
	iroN	AAGTCAAAGCAGGGGTTGCCCG	665	(8)
		GACGCCGACATTAAGACGCAG		

Supplementary Table 3. Clinical UPEC strain AT31 possesses the multidrug-resistant (MDR) phenotype. A total of 27 antibacterial agents in 12 categories were used, and the interpretation was done following the CLSI 2020 guideline. R ≥ 3 categories is considered MDR. S; susceptible. R; resistant.

Categories	Agent	AT31 status
Aminoglycosides	Gentamicin (CN)	S
	Tobramycin (TOB)	S
	Kanamycin (K)	S
	Amikacin (AK)	S
	Netilmicin (NET)	S
1st and 2nd generation cephalosporins	Cefazolin (KZ)	R
	Cefuroxime (CXM)	S
	Cefoxitin (FOX)	R
3rd and 4th generation cephalosporins	Cefotaxime (CTX)	S
	Ceftriaxone (CRO)	S
	Ceftazidime (CAZ)	S
	Cefepime (FEP)	S
Monobactams	Aztreonam (ATM)	S
Carbapenems	Doripenem (DOR)	S
	Ertapenem (ETP)	S
	Imipenem (IPM)	S
	Meropenem (MEM)	S
Tetracyclines	Tetracycline (TE)	S
	Doxycycline (DO)	S
Fluoroquinolones	Ciprofloxacin (CIP)	S
Folate pathway antagonist	Trimethoprim-Sulfamethoxazole (SXT)	R
Penicillins	Ampicillin (AMP)	R
Penicillins with β-lactamase inhibitors	Amoxycillin - Clavulanic Acid (AMC)	R
	Ampicillin – Sulbactam (SAM)	S
	Piperacillin - Tazobactam (TZP)	S
Phenicols	Chloramphenicol (C)	S
Fosfomycins	Fosfomycin (FOS)	S

Supplementary Table 4. qPCR primer list used in this study

Species	Gene	Sequence (5’->3’)	Reference
Mus musculus	*Gapdh*	TGTAGACCATGTAGTTGAGGTCA	(9)
		AGGTCGGTGTGAACGGATTTG	
	Nos2	CCAGCCTTGCATCCTCATTGG	
		CCAAACACCAAGCTCATGCGG	
	Il6	GCACAACTCTTTTCTCATTTCCACG	
		GCCTTCCCTACTTCACAAGTCCG	
	Tnfa	TTGGGTCTTGTTCACTCCACGG	
		CCTCTTTCAGGTCACTTTGGTAGG	
	Il10	GGTTGCCAAGCCTTATCGGA	(10)
		ACCTGCTCCACTGCCTTGCT	

Supplementary Table 5. Genome information of species closely related to our strain (AT31) and their accession number for calculating the ANI matrix and reconstructing the phylogenetic tree.

**Organism**	**Accession Number**
E. coli 55989	GCF_000026245.1
E. coli ABU83972	GCF_000148365.1
E. coli APEC O1	GCF_000014845.1
E. coli APEC O78	GCF_000332755.1
E. coli BL21 DE3	GCF_013167015.1
E. coli CFT073	GCF_014262945.1
E. coli O55H7 str. RM12579	GCF_000245515.1
E. coli SMS-3-5	GCF_000019645.1
E. coli str. K-12 substr. MG1655	GCF_000005845.2
E. coli UMN026	GCF_000026325.1
E. coli UTI89	GCF_000013265.1
Shigella boydii CDC 3083-94	GCF_000020185.1
Shigella flexneri 5-8401	GCF_000013585.1
Klebsiella pneumoniae HS11286	GCF_000240185.1
Proteus mirabilis HI4320	GCF_000069965.1

References for Supplementary Material

1. Wright KJ, Seed PC, Hultgren SJ. Uropathogenic Escherichia coli flagella aid in efficient urinary tract colonization. Infection and immunity. 2005;73(11):7657-68.

2. Mobley HL, Green DM, Trifillis AL, Johnson DE, Chippendale GR, Lockatell CV, et al. Pyelonephritogenic Escherichia coli and killing of cultured human renal proximal tubular epithelial cells: role of hemolysin in some strains. Infection and immunity. 1990;58(5):1281-9.

3. Nitisinprasert S, Nilphai V, Bunyun P, Sukyai P, Doi K, Sonomoto K. Screening and identification of effective thermotolerant lactic acid bacteria producing antimicrobial activity against Escherichia coli and Salmonella sp. resistant to antibiotics. Agriculture and Natural Resources. 2000;34(3):387-400.

4. Stojiljkovic I, Baumler AJ, Heffron F. Ethanolamine utilization in Salmonella typhimurium: nucleotide sequence, protein expression, and mutational analysis of the cchA cchB eutE eutJ eutG eutH gene cluster. J Bacteriol. 1995;177(5):1357-66.

5. Brons JK, Vink SN, de Vos MGJ, Reuter S, Dobrindt U, van Elsas JD. Fast identification of Escherichia coli in urinary tract infections using a virulence gene based PCR approach in a novel thermal cycler. J Microbiol Methods. 2020;169:105799.

6. Le Bouguenec C, Archambaud M, Labigne A. Rapid and specific detection of the pap, afa, and sfa adhesin-encoding operons in uropathogenic Escherichia coli strains by polymerase chain reaction. Journal of clinical microbiology. 1992;30(5):1189-93.

7. Yamamoto S, Terai A, Yuri K, Kurazono H, Takeda Y, Yoshida O. Detection of urovirulence factors in Escherichia coli by multiplex polymerase chain reaction. FEMS Immunol Med Microbiol. 1995;12(2):85-90.

8. Johnson JR, Russo TA, Tarr PI, Carlino U, Bilge SS, Vary JC, Jr., et al. Molecular epidemiological and phylogenetic associations of two novel putative virulence genes, iha and iroN(E. coli), among Escherichia coli isolates from patients with urosepsis. Infection and immunity. 2000;68(5):3040-7.

9. Winter SE, Winter MG, Xavier MN, Thiennimitr P, Poon V, Keestra AM, et al. Host-derived nitrate boosts growth of E. coli in the inflamed gut. Science (New York, NY). 2013;339(6120):708-11.

10. Xavier MN, Winter MG, Spees AM, Nguyen K, Atluri VL, Silva TM, et al. CD4+ T cell-derived IL-10 promotes Brucella abortus persistence via modulation of macrophage function. PLoS pathogens. 2013;9(6):e1003454.
