# Supplementary figures and images for: Probiotic Limosilactobacillus reuteri KUB-AC5 decreases urothelial cell invasion and enhances macrophage killing of uropathogenic Escherichia coli in vitro study
Source: Front Cell Infect Microbiol. 2024 Jul 18;14:1401462. doi: 10.3389/fcimb.2024.1401462 (PMC11291381; doi:10.3389/fcimb.2024.1401462)

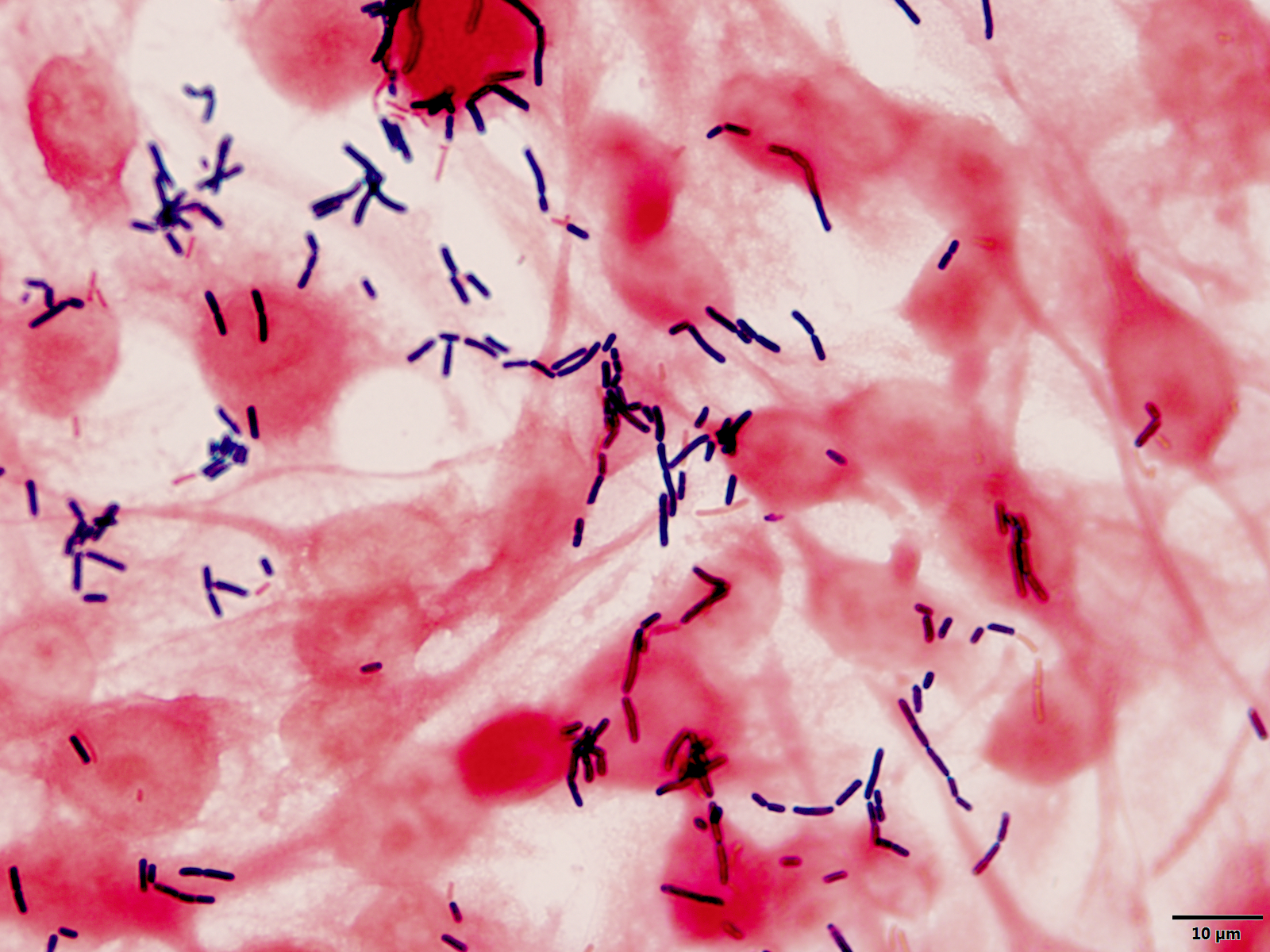

Supplement: Supplementary file 2 [file Image_2.tiff]

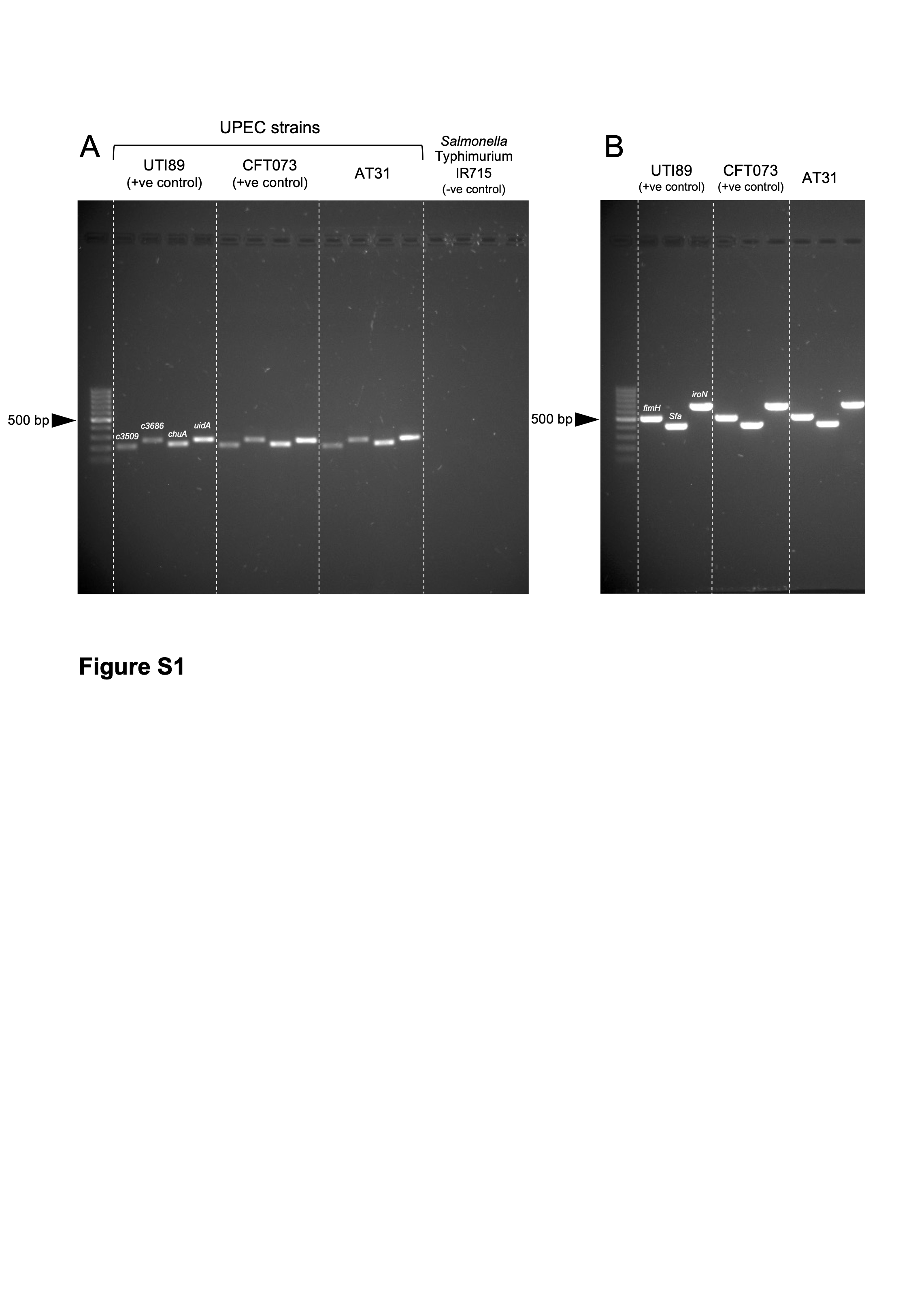

Supplement: Supplementary Figure 1 — Polymerase chain reaction confirmation of AT31 as a uropathogenic E. coli strain (1.2% agarose gel). Four UPEC signature genes (c3509, c3686, chuA, and uidA) were detected in AT31 (A). Three UPEC virulence genes (fimH, Sfa, and iroN) were detected in AT31 (B). [file Image_1.tiff]
